# Use of digital gene expression to discriminate gene expression differences in early generations of resynthesized *Brassica napus* and its diploid progenitors

**DOI:** 10.1186/1471-2164-14-72

**Published:** 2013-02-01

**Authors:** Jinjin Jiang, Yanlin Shao, Kun Du, Liping Ran, Xiaoping Fang, Youping Wang

**Affiliations:** 1 Jiangsu Provincial Key Laboratory of Crop Genetics and Physiology, Yangzhou University, Yangzhou 225009, China; 2 Oil Crops Research Institute, Chinese Academy of Agricultural Sciences, Wuhan 430062, China

## Abstract

**Background:**

Polyploidy is an important evolutionary mechanism in flowering plants that often induces immediate extensive changes in gene expression through genomic merging and doubling. *Brassica napus* L. is one of the most economically important polyploid oil crops and has been broadly studied as an example of polyploid crop. RNA-seq is a recently developed technique for transcriptome study, which could be in choice for profiling gene expression pattern in polyploids.

**Results:**

We examined the global gene expression patterns of the first four generations of resynthesized *B. napus* (F_1_–F_4_), its diploid progenitors *B. rapa* and *B. oleracea*, and natural *B. napus* using digital gene expression analysis. Almost 42 million clean tags were generated using Illumina technology to produce the expression data for 25959 genes, which account for 63% of the annotated *B. rapa* genome. More than 56% of the genes were transcribed from both strands, which indicate the importance of RNA-mediated gene regulation in polyploidization. Tag mapping of the *B. rapa* genome generated 19023, 18547, 24383, 20659, 18881, 20692, and 19955 annotated genes for the *B. rapa*, *B. oleracea*, F_1_–F_4_ of synthesized *B. napus*, and natural *B. napus* libraries, respectively. The unambiguous tag-mapped genes in the libraries were functionally categorized via gene ontological analysis. Thousands of differentially expressed genes (DEGs) were identified and revealed the substantial changes in F_1_–F_4_. Among the 20 most DEGs are DNA binding/transcription factor, cyclin-dependent protein kinase, epoxycarotenoid dioxygenase, and glycine-rich protein. The Kyoto Encyclopedia of Genes and Genomes (KEGG) analysis of the DEGs suggested approximately 120 biological pathways.

**Conclusions:**

The systematic deep sequencing analysis provided a comprehensive understanding of the transcriptome complexity of early generations of synthesized *B. napus*. This information broadens our understanding of the mechanisms of *B. napus* polyploidization and contributes to molecular and genetic research by enriching the *Brassica* database.

## Background

Polyploidization is an ancient and ongoing evolutionary process that promotes plant evolution by reshaping genomes [1,2]. The majority of flowering plants have undergone polyploidization (complete or partial) and chromosome rearrangement [3]. Generally, polyploids are divided into allopolyploids and autopolyploids [4,5]. Many major crops, including wheat, cotton, oat, coffee, and oilseed rape, are fundamentally allopolyploids [6-8]. Recently, many studies have revealed that methylation status, gene expression profile, chromosomal rearrangements, deletions, insertions, and sequence substitutions in many allopolyploids differ to their progenitor [9,10]. Birchler and Veitia [11] proposed the gene balance theory with regard to quantitative traits and gene duplication following polyploidy [11]. Angiosperm genome plasticity in polyploids is always related to changes in gene expression, which are largely controlled by epigenetic profiles [12,13]. Gaeta and Pires [14] concluded that these genomic changes occur in both natural and resynthesized polyploids [14]. Molecular analyses of natural and resynthesized allopolyploids indicate that genetic and epigenetic changes are common results of polyploidization in different species [5,15,16]. Discovering the structural and functional evolution of genomes during polyploidization is of great importance in plant biology [17].

Cultivated *Brassica* species include important economical crops mostly closely related to *Arabidopsis thaliana* and provide great chances for studying the effects of polyploidization [3]. The availability of the *Arabidopsis* genome effectively facilitates studies on *Brassica* polyploidization [18]. Soon after the *Arabidopsis* and *Brassica* lineage diverged ~17.0 million years ago (Mya), the triplicated *Brassica* subgenomes divergence was estimated to be 14.3 Mya [17,19]. *B. rapa* (A genome) and *B. oleracea* (C genome) descended from a common hexaploid ancestor of *A. thaliana*. Moreover, the lineage of *B. rapa* and *B. oleracea* diverged around 3.5 Mya [20], and a recent segmental duplication in *B. rapa* occurred ~0.8 Mya [21]. Studies on A- and C-genome mapping, genome comparison, and genome evolution have been performed during the past decades. Additional genome duplication aside from triplication, as well as complex chromosomal changes was revealed in *B. oleracea*[22,23]. *B. rapa*, ~529 Mb per haploid, was first launched for complete genome sequencing. The allopolyploid *B. napus* (AC, n = 19) was spontaneously derived from hybridization of A and C progenitors [24]. The genome of natural *B. napus* was confirmed intact without rearrangement, but resynthesized *B. napus* underwent rapid changes in the early generations, including genetic changes and methylation changes [25-28]. Non-additive proteins and additive proteins were completely identified in resynthesized *B. napus*, indicating the early steps of allopolyploidization repatterning are controlled by nonstochastic mechanisms [10,29]. Xiong et al. [30] indicated that aneuploidy, gross chromosomal rearrangements, and dosage balance maintain the genomic stability of synthesized *B. napus*[30].

Considering microarrays enable the comparison of gene expression at the transcriptome level, a set of *Brassica* unigenes assembled using *Brassica* expressed sequence tags (ESTs) was developed and assigned to discriminate paralogous genes, but not homologous genes, between the A- and the C-genome [6,31]. Recently emerged next generation sequencing (NGS) technology is an alternative method for better genome, epigenome, and transcriptome study [32,33]. Many plant species have benefited from this technology, including *B. rapa* and *B. napus*. The draft genome sequence of *B. rapa* accession Chiifu-401-42 was newly released by Illumina GA II technology and annotated [34], which provides an important resource for studying the evolution of polyploid genomes. Leaf transcriptome of *B. napus* had been dissected by sequencing [35,36].

In the present research, we conducted a digital gene expression (DGE) analysis on resynthesized *B. napus* across the F_1_–F_4_ generations to address the transcriptome changes after polyploidization, which were also compared with their genetic progenitors (*B. rapa* and *B. oleracea*) and natural *B. napus*. Previous studies on proteomic changes in this population revealed that differentially expressed proteins in F_1_ differed from the progenitors, exhibiting non-additive repatterning. Furthermore, gene silencing during polyploidization induces differences in protein expression in different generations of synthesized *B. napus*[37]. We report the expression profile of genes in resynthesized *B. napus*, show the upregulation of essential pathways and genes in F_1_, and compare them with progenitors, which are downregulated in the F_2_–F_4_ generations. This is the first comprehensive transcriptomic research that identifies DEGs and the pathways involved in first four generations of synthesized *B. napus* after polyploidization.

## Results

### Digital gene expression (DGE) profile

**Figure 1:**
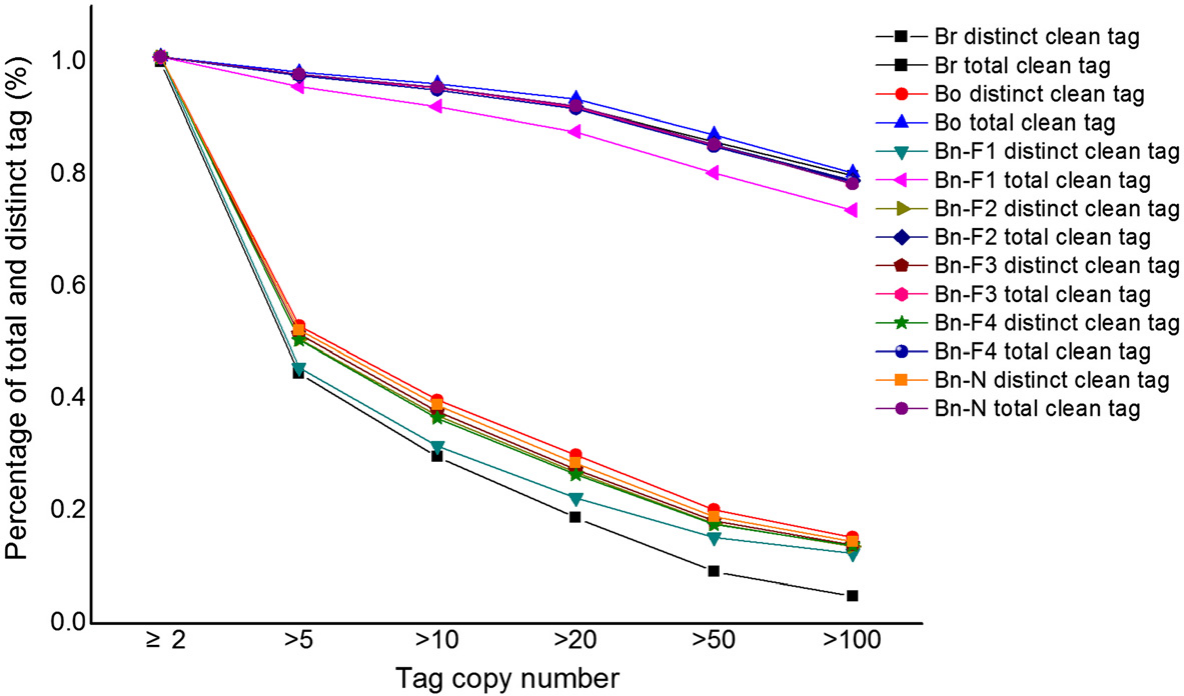
Distribution of total tag and distinct tag counts over different tag abundance categories from the seven libraries.

To investigate global transcriptome during the polyploidization of *B. napus*, we performed a DGE analysis on the seedling stage of resynthesized *B. napus* (F_1_–F_4_) and its diploid parents, as well as natural *B. napus*. Finally, DGE libraries from the leaves of four-week-old plants were generated and sequenced by Illumina technology. The sequence data are available from the GEO repository, accession number GSE43246. The statistics of the DGE tags is shown in Table 1. Approximately six million raw tags were generated for each library, and more than 97% of the raw tags were clean tags. A total of 6178564 raw tags were obtained from *B. rapa* (Br), 6059222 from *B. oleracea* (Bo), 6155227 from *B. napus-*F_1_ (Bn-F_1_), 6092805 from *B. napus-*F_2_ (Bn-F_2_), 6142098 from *B. napus-*F_3_ (Bn-F_3_), 5938583 from *B. napus-*F_4_ (Bn-F_4_), and 5964594 from natural *B. napus* (Bn-N). After removing the low-quality sequences and adapter sequences, 6018254, 5930726, 6022170, 5950123, 5991210, 5798939, and 5823113 clean tags were obtained with 21 nt in length in the corresponding species. Unambiguous tags were counted and normalized to the number of transcripts per million tags (TPM) to evaluate the gene expression level. The results show that the mRNA transcribed from major genes had fewer than ten copies and only a small proportion of genes were highly expressed. The distribution of clean tags in the seven libraries was determined to evaluate the normality of the dataset. The results show a consistent pattern, with most of the tags coming from highly expressed genes. In this analysis, the total number of clean tags is the sum of all clean tags and the number of distinct clean tags is the number of different clean tags (Figure 1, Additional file 1: S1 and Additional file 2: S2). We found that the percentage of distinct tags with high counts dropped dramatically, and the distinct tags with more than 100 copies accounted less than 10%. However, more than 70% of total clean tags have accounts above 100 in each library. By contrast, 50% of the distinct clean tags had copy numbers between two and five, but they only represented around 4% of the total number of clean tags, which indicates only a small number of mRNA were expressed at high abundance and the majority were expressed at very low levels [38]. The clean tags were then mapped onto the *B. rapa* genome [34] and the numbers of tags that could be mapped onto genes with a maximum of one base pair mismatch in Br, Bo, Bn-F_1_, Bn-F_2_, Bn-F_3_, Bn-F_4,_ and Bn-N were 1964909, 1747843, 3025405, 2354059, 1652733, 2295897, and 2253347, respectively (Table 1). Statistical analysis of clean tag alignment was conducted, including analysis of total clean tags and distinct clean tags (Additional file 3: S3, Additional file 2: S2). We found that around 70% of total clean tags were mapped onto the *B. rapa* genome with perfect match or with 1 bp mismatch to sense or anti-sense genes, and approximately 65% of the distinct clean tags were successfully mapped. Finally, the tag mapping onto the *B. rapa* genome generated 19023 tag-mapped genes for Br, 18547 for Bo, 24383 for Bn-F_1_, 20659 for Bn-F_2_, 18881 for Bn-F_3_, 20692 for Bn-F_4_, and 19955 for Bn-N. In total, 25959 genes were identified from the seven libraries, which accounted for approximately 63% of genes in the annotated *B. rapa* genome (Additional file 4: S4).

**Table 1:** Statistics of categorization and abundance of DGE tags

**Summary**		***B. rapa***	***B. oleracea***	***B. napus-*F**_**1**_	***B. napus-*F**_**2**_	***B. napus-*F**_**3**_	***B. napus-*F**_**4**_	***B. napus-*N**
Raw Data	Total	6178564	6059222	6155227	6092805	6142098	5938583	5964594
Raw Data	Distinct Tag	293575	243895	331406	275597	284202	272986	269285
Clean tag	Total number	6018254	5930726	6022170	5950123	5991210	5798939	5823113
Clean tag	Distinct Tag number	133499	116771	199428	134403	134721	134857	128967
Tag Mapping to Gene	Total number	1964909	1747843	3025405	2354059	1652733	2295897	2253347
Tag Mapping to Gene	Distinct Tag number	44267	36220	88270	49781	42613	51122	45358
Unambiguous Tag Mapping to Gene	Total number	1679848	1475050	2495411	2016182	1415736	1971591	1924944
Unambiguous Tag Mapping to Gene	Total % of clean tag	27.91%	24.87%	41.44%	33.88%	23.63%	34.00%	33.06%
Unambiguous Tag Mapping to Gene	Distinct Tag number	39414	31933	79648	44456	37996	45762	40561
Unambiguous Tag Mapping to Gene	Distinct Tag % of clean tag	29.52%	27.35%	39.94%	33.08%	28.20%	33.93%	31.45%
Tag-mapped Genes	number	19023	18547	24383	20659	18881	20692	19955
Tag-mapped Genes	% of ref genes	46.20%	45.05%	59.22%	50.17%	45.86%	50.26%	48.47%
Unambiguous Tag-mapped Genes	number	16574	15970	22059	18155	16479	18196	17448
Unambiguous Tag-mapped Genes	% of ref genes	40.25%	38.79%	53.58%	44.09%	40.02%	44.19%	42.38%
Mapping to Genome	Total number	2437918	2105332	1776978	2290544	2658991	2232572	2164464
Mapping to Genome	Total % of clean tag	40.51%	35.50%	29.51%	38.50%	44.38%	38.50%	37.17%
Mapping to Genome	Distinct Tag number	44076	30703	49898	42334	45680	42590	40689
Mapping to Genome	Distinct Tag % of clean tag	33.02%	26.29%	25.02%	31.50%	33.91%	31.58%	31.55%
Unknown Tag	Total number	1615427	2077551	1219787	1305520	1679486	1270470	1405302
Unknown Tag	Total % of clean tag	26.84%	35.03%	20.25%	21.94%	28.03%	21.91%	24.13%
Unknown Tag	Distinct Tag number	45156	49848	61260	42288	46428	41145	42920
Unknown Tag	Distinct Tag % of clean tag	33.82%	42.69%	30.72%	31.46%	34.46%	30.51%	33.28%

Clean tags are tags after filtering dirty tags from raw data. Distinct tags are different kinds of tags and unambiguous tags are the reminder clean tags after removing tags mapped to reference genome.

To determine whether the sequencing depth was sufficient for the transcriptome coverage, a saturation analysis was performed to check whether the number of detected genes increased with sequencing amount until the number of reads reached 2 million (Additional file 5: S5). We also analyzed the distribution of the ratio of distinct tag copy numbers in each pair of libraries and found that more than 90% of the distinct tags had ratios up to 5-fold (Additional file 6: S6). Antisense genes play an important role in gene expression and regulation, and about 36% (14775) of the genes were matched by distinct tags in antisense orientations. Up to 5726 genes were mapped by DGE tags in both sense and antisense orientations in the Br library, 5123 in the Bo library, 11903 in the Bn-F_1_ library, 6568 Bn-F_2_, 5692 in the Bn-F_3_ library, 6887 in the Bn-F_4_ library, and 6253 in the Bn-N library (Additional file 4: S4). In total, more than 56% of the genes (14775 of 25959 genes) were transcribed from both strands, which indicate the importance of RNA-mediated gene regulation in polyploidization.

### Differentially expressed genes in early generations of resynthesized *B. napus*

**Figure 2:**
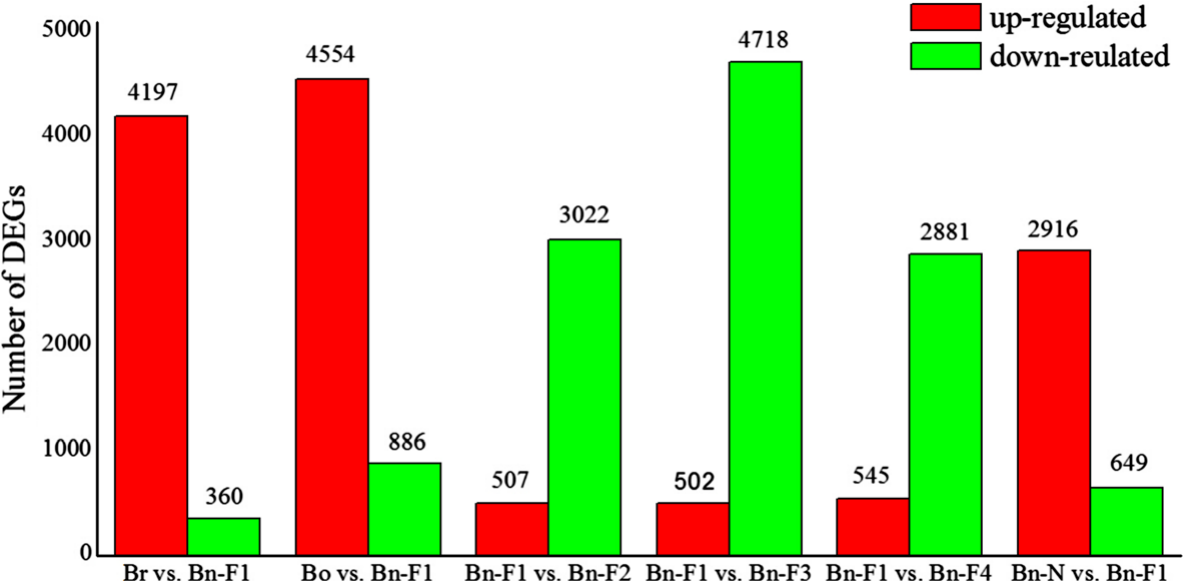
**Numbers of differentially expressed genes in each comparison.** The numbers of up-regulated (in red) and down-regulated genes (in green) are presented. ‘A’ was the control and ‘B’ was experimental group in ‘A vs. B’.

**Figure 3:**
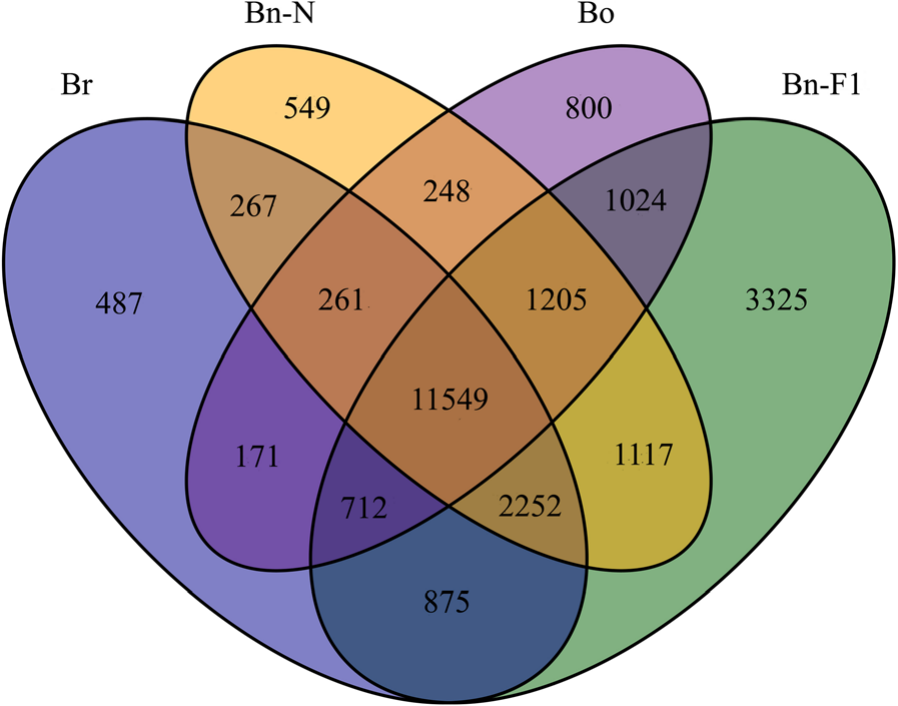
**Distribution of the genes commonly and specifically expressed in natural *B. napus*, synthesized *B. napus*-F**_**1 **_**and its progenitors (*B. rapa *and *B. oleracea*).**

**Figure 4:**
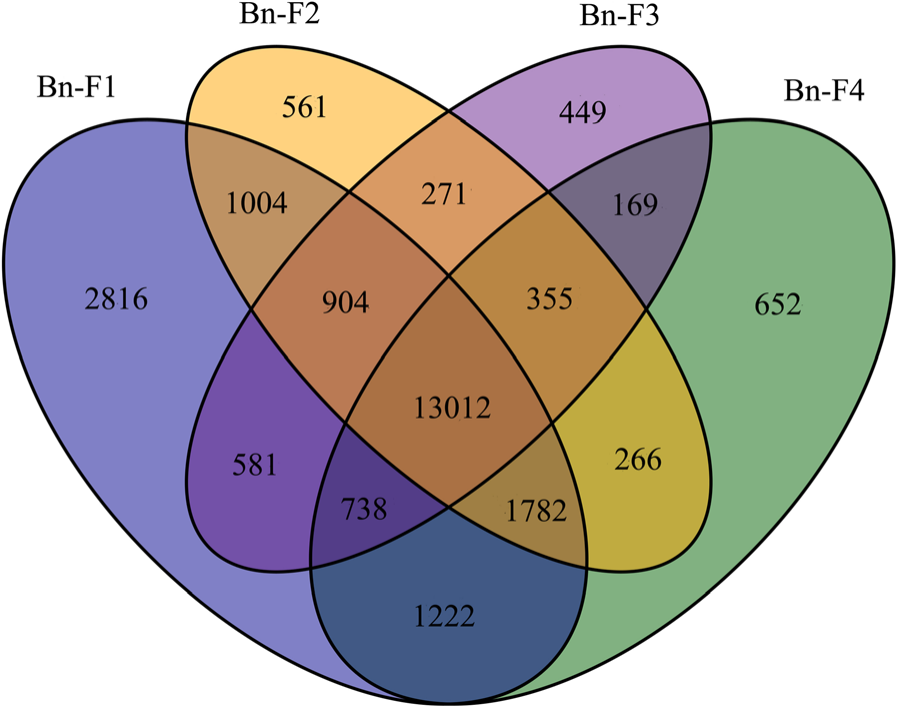
**Distribution of the genes commonly and specifically expressed in synthesized *B. napus*-F**_**1**_**, *B. napus*-F**_**2**_**, *B. napus*-F**_**3 **_**and *B. napus*-F**_**4.**_

To identify genes that were differentially expressed in the early generations of synthesized *B. napus* after polyploidization, we compared pairs of DGE profiles of the seven libraries (Br versus Bn-F_1_, Bo versus Bn-F_1_, Bn-F_1_ versus Bn-F_2_, Bn-F_1_ versus Bn-F_3_, Bn-F_1_ versus Bn-F_4_, and Bn-N versus Bn-F_1,_ where A was the control and B was experimental group in ‘A versus B’) to analyze gene expression variations (Figure 2 and Additional file 7: S7). The comparison of *B. napus-*F_1_ with *B. rapa* revealed that 4197 DEGs were significantly upregulated and 360 DEGs were downregulated in *B. napus-*F_1_ in comparison with *B. rapa*. By contrast, 4554 DEGs were downregulated and 886 DEGs were upregulated in *B. napus-*F_1_ compared with *B. oleracea*. The number of upregulated DEGs in *B. napus*-F_1_ was more than that downregulated after polyploidization, which might indicate heterosis. Apart from the DEGs among resynthesized *B. napus*-F_1_ and its progenitors, the number of the identified DEGs differed in the early generations of synthesized *B. napus*, and most of the DEGs showed downregulation in the F_2_–F_4_ generations compared with the F_1_ generation. Up to 3022 DEGs were downregulated and 507 were upregulated in *B. napus-*F_2_ compared with *B. napus-*F_1_; 4718 DEGs were downregulated and 502 were upregulated in *B. napus-*F_3_ compared with *B. napus-*F_1_; 2882 DEGs were downregulated and 545 DEGs were upregulated in *B. napus-*F_4_ and *B. napus-*F_1_. Comparison of resynthesized and natural *B. napus* showed 649 tags were downregulated and 2916 tags were upregulated in *B. napus-*F_1_ in comparison with natural *B. napus*. By comparison, the lowest number of DEGs was found in the F_4_ generation, which was in a relatively stable stage after polyploidization. Figure 3 shows the distribution of genes commonly expressed in *B. rapa*, *B. oleracea*, *B. napus*-F_1_, and natural *B. napus*, which indicates that a number of conserved genes were identified apart from the DEGs. Distribution of genes commonly and specifically expressed in Bn-F_1_, F_2_, F_3_ and F_4_ was also analyzed (Figure 4). Among the DEGs, the 20 most abundantly expressed genes that were upregulated or downregulated during polyploidization are listed in Additional file 8: S8. We found that the genes that encode DNA binding/transcription factor (Bra039065, Bra018796) were more prominent, which were downregulated in F_1_ compared with its progenitors and then upregulated in the F_2_–F_3_ generations compared with the F_1_ generation. Genes encoding cyclin-dependent protein kinase (Bra016640), epoxycarotenoid dioxygenase (Bra020970), and glycine-rich protein (Bra040817) were upregulated in F_1_ during polyploidization, and then downregulated in F_2_–F_4_ compared with F_1_**.**

### Functional annotation of DEGs

**Figure 5:**
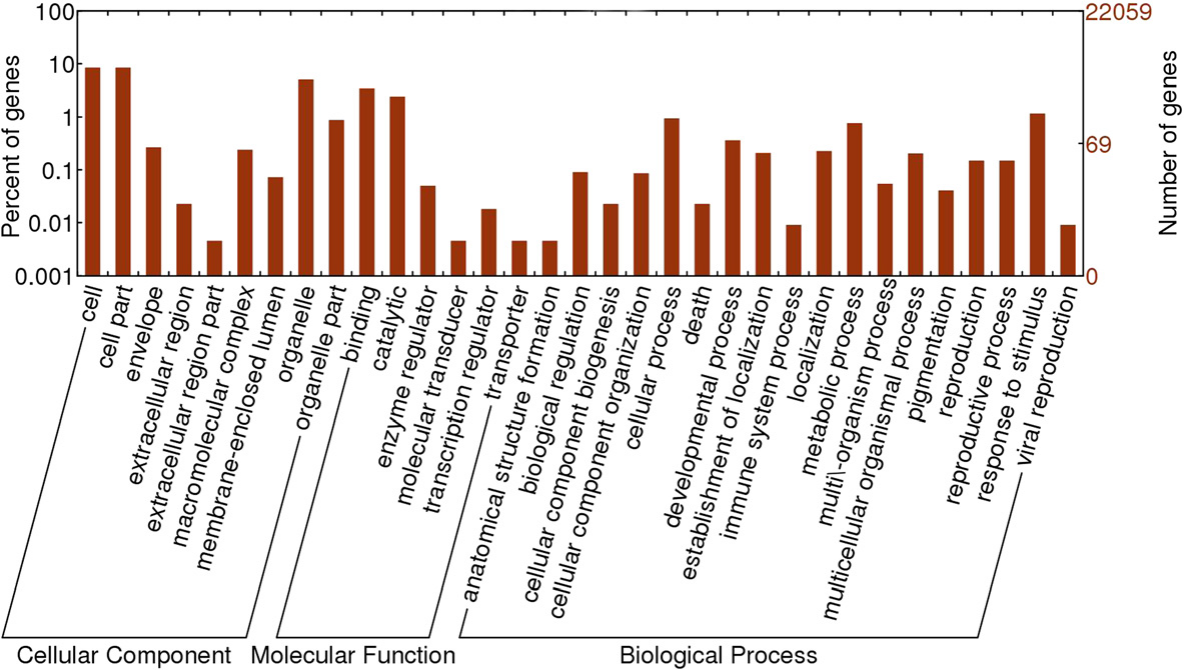
**Histogram presentation of gene ontology classification of synthesized *B. napus*-F**_**1**_**.** 22059 unambiguously mapped genes were assigned to three main categories: cellular component, biological process and molecular function. The right- and left-axis indicates the number of genes in a category and the percentage of the specific category of genes in the main category, respectively.

Annotation of the sequences using GO database yielded good results for 22059 unambiguously mapped genes identified using the Bn-F_1_ DGE tags. These well-annotated sequences belonged to three main categories (cellular component, molecular function, and biological process) and distributed into 33 categories, including the most dominant pathways such as ‘binding and catalytic activity,’ ‘cell and cell part,’ ‘organelle,’ ‘cellular and metabolic processes,’ and ‘response to stimulus’ (Figure 5). A similar GO distribution was revealed in the other six libraries (Additional file 9: S9). However, we did not find any genes in the transporter cluster in Bo. Genes were not found in the extracellular region cluster in Bn-F_2_. Enzyme regulators and translation regulators were not observed in Bn-F_3_.

To identify the biological pathways active in the DGE libraries, we mapped all the annotated genes to terms in the KEGG database to search for significantly enriched genes involved in metabolic or signal transduction pathways (Additional file 10: S10). Among all the genes with KEGG annotation, DEGs were identified in Bn-F_1_ in comparison with Br. In total, we assigned 2911 DEGs to 121 KEGG pathways. Up to 38 of these pathways were significantly enriched, with Q values ≤ 0.05 (red border region), including metabolic pathways, ribosome, and carbon fixation in photosynthetic organisms. Similar pathway enrichment was revealed in pair comparison of each libraries (Bo vs. Bn-F_1_, Bn-F_1_ vs. Bn-F_2_, Bn-F_1_ vs. Bn-F_3_, Bn-F_1_ vs. Bn-F_4_, and Bn-N vs. Bn-F_1,_ where A was the control and B was experimental group in ‘A vs. B’) . The 3339 DEGs identified in Bn-F_1_ in comparison with Bo were assigned to 122 KEGG pathways, 44 of which were significantly enriched. The 2293 DEGs identified in Bn-F_2_ in comparison with Bn-F_1_ were assigned to 122 KEGG pathways, 3322 DEGs identified in Bn-F_3_ in comparison with Bn-F_1_ were assigned to 120 pathways, 2229 DEGs identified in Bn-F_4_ in comparison with Bn-F_1_ were assigned to 119 pathways, and 2283 DEGs identified in Bn-F_1_ in comparison with Bn-N were assigned to 119 pathways.

### Genome-wide non-additive gene regulation in synthesized Bn-F_1_

We compared the transcript abundance in synthesized Bn-F_1_ with the relative mid-parent value (MPV: an equal mixture of RNA from two parents) to identify genes that showed differential expression pattern in Bn-F_1_. Up to 19785 genes in Bn-F_1_ showed differences in expression from MPV, 12012 (60.7%) of them showed higher expression levels than MPV, and 7773 (39.3%) of these genes showed lower expressions than MPV. Among these non-additively expressed genes, 9456 (47.8%) genes were expressed at higher levels in Br than in Bo, and 10329 (52.2%) genes were expressed at lower levels in Br than Bo (Table 2). The data suggest that the orthologous genes in polyploids are frequently expressed in a non-additive pattern.

**Table 2:** **Number of non-additively expressed genes in resynthesized Bn-F**_**1**_

	**a**	**%**	**b**	**%**	**b/a (%)**	**c**	**%**	**c/a (%)**
	**No. of non-additively expressed genes**		**No. of non-additively expressed genes**			**No. of non-additively expressed genes**		
	**Hybrid versus MPV**		**Hybrid > MPV**			**Hybrid < MPV**		
Bn-F_1_	19785		12012		60.7	7773		39.3
Br > Bo	9456	47.8	6134	51.1	64.9	3322	42.7	35.1
Br < Bo	10329	52.2	5878	48.9	56.9	4451	57.3	43.1

### Cluster analysis of DEGs

**Figure 6:**
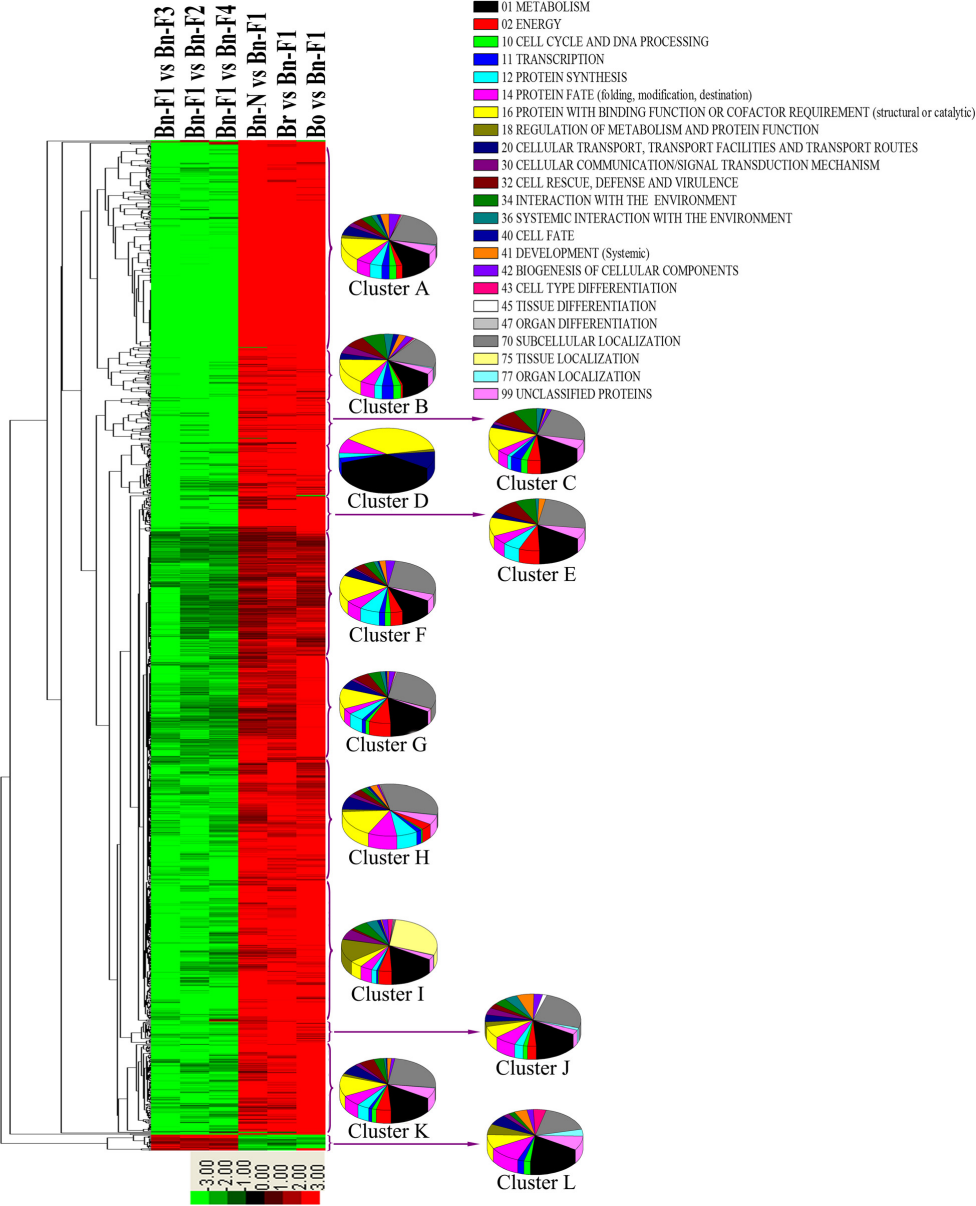
**Hierarchical cluster analysis of differentially expressed transcripts between each comparisons (Bn-F**_**1 **_**vs. Bn-F**_**3**_**, Bn-F**_**1 **_**vs. Bn-F**_**2**_**, Bn-F**_**1 **_**vs. Bn-F**_**4**_**, Bn-N vs. Bn-F**_**1**_**, Br vs. Bn-F**_**1**_**, Bo vs. Bn-F**_**1**_**.** ‘A’ was the control and ‘B’ was experimental group in ‘A vs. B’). The letters from A to L indicates the twelve major clusters resulted from HCE analysis. Pie charts represent functional classification of DEGs in each cluster based on MIPS functional catalogue.

Cluster analysis of DEGs in the early generations of synthesized *B. napus* was performed with the correlated expression profile (Additional file 4: S4). Generally, 7948 DEGs in all the comparisons were clustered as the union of DEGs. Up to 1358 DEGs in all the comparisons were clustered as the DEG intersections (Figure 6). Among the 12 major clusters, the upregulated transcripts were enriched in clusters A–K in the comparisons of Bn-N vs. Bn-F_1_, Br vs. Bn-F_1_, and Bo vs. Bn-F_1_. The downregulated transcripts were enriched in clusters A–K in the comparisons of Bn-F_1_ vs. Bn-F_2_, Bn-F_1_ vs. Bn-F_3_, and Bn-F_1_ vs. Bn-F_4_. Only one exception indicated that cluster L showed gene upregulation in Bn-F_2,_ Bn-F_3_ and Bn-F_4_ compared with Bn-F_1_, and downregulation in Bn-F_1_ compared with Bn-N, Br and Bo. We also identified the enrichment of DEGs in each cluster in different functional categories and found that the genes involved in the metabolism category were enriched significantly in all clusters except cluster H. Those in the protein with binding function or cofactor requirement category were enriched significantly in clusters A–H, whereas those in the subcellular localization category were enriched in all clusters except for cluster D. All the clustered DEGs are listed in Additional file 11: S11. Multiple genes represented for proteins with binding function, such as zinc ion binding (Bra019896), ATP binding (Bra008602, Bra023518), glutathione binding (Bra022815), selenium binding (Bra032756), and FAD/NADP/NADPH binding (Bra001931), showed different expression values in different generations of resynthesized *B. napus* and its progenitors. Some genes that encode ribosomal proteins were also changed during polyploidization.

## Discussion

### Differences in gene expression between synthesized *B. napus*-F_1_ and its progenitors

Based on analysis of the leaf transcriptome data for synthesized *B. napus*-F_1_ and its progenitors, as well as the natural *B. napus* generated in this study, we found that the majority of the *B. napus*-F_1_ transcripts were conserved with the parental sequences. Up to 19023, 18547, and 24383 sequences were mapped by *B. napus*-F_1_ and its progenitors *B. rapa* and *B. oleracea*. 3182 genes with same TPM were expressed in Bn-F_1_ and Br, and 2421 genes with same TPM were expressed in Bn-F_1_ and Bo, which indicate that these genes might be significantly inherited by Bn-F_1_ from one of its progenitors. The genes with different TPM expressed in *B. napus*-F_1_ and its progenitors, which indicate that the genome combination results duplicated genes in the resynthesized polyploid. However, only 14 of these commonly expressed genes showed TPM values in Bn-F_1_ equal to the combination of its progenitors. Furthermore, most of the commonly expressed genes in the progenitors were non-additively expressed in Bn-F_1_, which might be responsible for gene repression and activation [5,28,39]. The majority of the non-additively expressed genes in Bn-F_1_ displayed expression values different to its progenitors, indicating that the transcriptome was reconciled during polyploidization [40]. Non-additive gene regulation should be involved in various biological pathways, which may lead to subfunctionalization of duplicated genes [41]. The gene expression in the resynthesized Bn-F_1_ was more complicated than the simple combination of two genomes, which might be due to the homologous recombination between closely related genomes (A- and C-genome) [42]. Xiong et al. [30] reported the variable chromosome instability of synthesized *B. napus*, and chromosome loss was compensated by the gain of homologous chromosomes [30]. Szadkowski et al. [43] reported the genome blender of synthesized *B. napus* during first meiosis, which resulted in the rearrangement of two genomes and the restructuring in further generations [43]. Thus, resynthesized polyploids derived from closely related species result in changes in the non-additive pattern of multiple gene expression, which explains the molecular bases for hybrid vigor [44].

Besides the commonly expressed genes in polyploids and diploid progenitors, there are some species specific genes. As shown in Figure 3, 487, 800, 3325 and 549 genes were specifically expressed in Br, Bo, Bn-F_1_ and Bn-N, respectively. The number of Bn-F_1_ specific genes was much higher than its progenitors, indicating many new transcripts emerged during polyploidization. This was consistent with Xu et al. [45] that 20 novel transcripts were detected in leaves of resynthesized Bn-F_1_[45]. Based on the clustering analysis of DEGs, we found 1333 out of the 1358 interspecific DEGs were up-regulated in Bn-F_1_ compared with Br and Bo. These up-regulated genes express proteins with different biological function, including salt tolerance protein, mitochondria/chloroplast membrance binding proteins, kinds of protein kinases, etc. Confirmation of these expression differences in further study is necessary for deep into the genetic causes of interest characteristics and improved qualities. Whereas 18 out of the 1358 interspecific DEGs were down-regulated in Bn-F_1_ compared with Br and Bo. Only two genes (Bra005226 and Bra026817) were up-regulated in Bn-F_1_ compared with Br, whereas down-regulated in Bn-F_1_ compared with Bo. Five genes (Bra040268, Bra038457, Bra033201, Bra037320 and Bra039516) were up-regulated in Bn-F_1_ compared with Bo, whereas down-regulated in Bn-F_1_ compared with Br (Figure 6). All these gene expression differences are of great interest for studying the genome polyploidization of *Brassica* species.

### Gene expression differences among early generations of synthesized *B. napus* (F_1_-F_4_) and natural *B. napus*

Hitherto, studies on polyploidy mechanism were conducted on natural and resynthesized *B. napus* using proteomic, transcriptome, and cytogenetic analyses [6,10,29,31,39,43]. However, few studies that trace genomic changes (including cytosine methylation, DNA fragment loss, genetic rearrangement) and transcriptome changes in different generations of synthesized *B. napus* have been reported [9,27,28]. As mentioned above, many novel transcripts emerged in Bn-F_1_ compared its progenitors. The number of Bn-F_1_ specific genes was also higher than Bn-F_1_, Bn-F_2_, Bn-F_3_ (Figure 4). Many of these new transcripts emerged in the first generation of resynthesized *B. napus* disappeared after successive self hybridizations, indicating the instability of resynthesized polyploids. Based on the clustering of DEGs found in the comparisons of Br vs. Bn-F_1_, Bo vs. Bn-F_1_, Bn-F_1_ vs. Bn-F_2_, Bn-F_1_ vs. Bn-F_3_, Bn-F_1_ vs. Bn-F_4_, and Bn-N vs. Bn-F_1_, we easily found that most DEGs are significantly downregulated in Bn-F_2_, Bn-F_3_, and Bn-F_4_ compared with Bn-F_1_, but these DEGs were upregulated in Bn-F_1_ compared with its progenitors and natural *B. napus*. This phenomenon also indicated the genomic instability after polyploidization, and the discovery of multiple gene expression differences may compensate for the limitation of proteomic analysis [37]. The upregulation of DEGs in synthesized Bn-F_1_ during polyploidization could explain its improved characteristics compared with its progenitors. For example, the expression pattern of Bra022585, which encodes stress-responsive protein and Bra040633, which encodes defense-related protein, were changed during polyploidization. Some of these gene expression differences might be related to the DNA methylation or gene fragment losses during the combination of two genomes [45]. For example, Bra003009, which encodes O-methyltransferase 1, and Bra006323, which encodes an N2-dimethylguanosine tRNA methyltransferase family protein, were upregulated in Bn-F_1_ during polyploidization and downregulated in the F_2_–F_4_ generations. In the pathway analysis, we found Bra003009 participated in the flavone and flavonol biosynthesis, which functioned in the transformation of kaempferide into kaempferol. This may indicate that kaempferol level was different in the synthesized *B. napus*. The changes in expression include many transcription factors, binding proteins, and ribosomal proteins, must be important for the merging of the A- and C-genomes in synthesized polyploids. For the 18 DEGs, which were down-regulated in Bn-F_1_ compared with Br and Bo, we found they were up-regulated in Bn-F_2_, Bn-F_3_ and Bn-F_4_ compared with Bn-F_1_. This indicated that transcriptome recovery of genome balance functioned after polyploidization. Further research is needed to verify and determine the causes of these transcriptome changes and how they correlate with the phenotypic divergence and fertility of synthesized polyploids.

## Conclusions

By applying the DGE deep sequencing, this study investigated the transcriptome profile of resynthesized *B. napus* and its diploid progenitors, as well as natural *B. napus*, with an aim to illustrate the gene expression differences during polyploidization. The amounts of transcripts obtained provided a foundation for future research on polyploidy mechanism of *B. napus*. Globally identified DEGs and putative biological pathways revealed the gene expression difference between synthesized *B. napus* and progenitors, and differential expression of genes across generations of synthesized *B. napus*. Generally, the gene expression in the resynthesized *B. napus* was more complicated than the simple combination of two genomes, and non-additive gene regulation was also detected in synthesized *B. napus*_._ These findings provided a contribution to the existing sequence resources for *Brassica* and would certainly facilitate polyploidy research of this genus.

## Methods

### Plant material

Seeds of resynthesized *B. napus* (F_1_ generation) and its successive selfing generations (F_2_-F_4_), its diploid progenitors *B. rapa* (cv. Aikangqing) and *B. oleracea* (cv. Zhonghua Jielan), were generously provided by the Oil Crops Research Institute of the Chinese Academy of Agricultural Sciences. Natural *B. napus* cv. Yang6 was provided by the Jiangsu Institute of Agricultural Science in the Lixiahe District (China). All the plants were cultivated in climate chambers at 25°C, a 16 h light: 8 h dark photoperiod, and 70% relative humidity. The first true leaves from three plants of each genotype were pooled at the same physiologic stage (28-day-old seedlings) and frozen at 80°C for use.

### RNA preparation, Illumina RNA-sequencing and data processing

Total RNA was extracted from leaves using RNAiso Plus (Takara) according to the manufacture’s protocol. RNA concentrations were measured using a Qubit Fluorometer, and integrity was confirmed via 2100 Bioanalyzer (Agilent Technologies). The DGE libraries were prepared using Illumina Gene Expression Sample Prep Kit. Single-chain molecules were fixed onto a Solexa Sequencing Chip (flowcell) and sequenced by Illumina HiSeq™ 2000 System. Millions of raw, 35 bp sequences were generated. Image analysis, base calling, generation of raw tags, and the tags were counted using the Illumina pipeline [38]. Empty tags (no tag sequence between the adaptors), adaptors, low quality tags (tags containing one or more unknown nucleotides “N”), and tags with a copy number of 1 were removed from raw sequences to obtain clean tags (21 bp) containing CATG.

### Mapping of reads to the reference sequence

To identify the gene expression patterns in early generations of synthesized *B. napus*, all clean tags were annotated by mapping to the sequenced genome of *B. rapa*[34] using the SOAP2 software, with a maximum of 1 nucleotide mismatch allowed [46]. All the tags mapped to reference sequences were filtered and the remaining tags were designated as ambiguous tags. Mapping events on both sense and antisense sequences were included in the data processing. For gene expression analysis, the number of expressed tags was calculated and then normalized to TPM (number of transcripts per million tags) [47,48]; and the DEGs were screened and used for mapping and annotation [49,50]. Gene annotation was conducted using Blast2GO [51]. The gene ontology (GO) categorization of all DEGs was displayed as three hierarchies for cellular component, molecular function, and biological process by searching in the GO database. Web Gene Ontology Annotation Plot (WEGO) was also used for GO classification of genes mapped by each DGE library [52]. Clustering analysis of differential gene expression pattern was also conducted using hierarchical clustering explorer (HCE) [53,54]. In this study, statistical analysis of DEGs among libraries was performed using stringent value FDR ≤ 0.001 (false discovery rate) and the absolute value of |log2Ratio| ≥ 1 as the threshold of significant difference of gene expression. The DEGs in the hierarchical clustering were grouped into functional categories based on MIPS functional catalogue using *Arabidopsis* orthologues [55]. Pathway enrichment analysis of differential gene expression was conducted for further understanding gene function through blasting the KEGG database. A P-value of 0.05 was selected as the threshold for considering a gene set as significantly enriched.

## Competing interests

The authors declare that they have no competing interests.

## Authors’ contributions

YW conceived and designed the study. JJ, YS and KD participated in the experiments. JJ, LR and XF analyzed the data. All authors drafted the manuscript and approved the final manuscript.

## Supplementary Material

Additional file 1: S1 Distribution of total clean tags and distinct clean tags over different tag abundance categories in each DGE library. (**A**) Distribution of total tags. Numbers in the brackets of indicate the range of copy numbers for a specific category of tags. For example, [2,5] means all the tags in this category has 2 to 5 copies. Numbers in the parentheses show the total tag copy number for all the tags in that category. (**B**) Distribution of distinct tags. Numbers in the square brackets indicate the range of copy numbers for a specific category of tags. Numbers in the parentheses show the total types of tags in that category. Click here for file

Additional file 3: S3 Mapping results of total tags and distinct tags of species in seven libraries. Normalized tag copy number was calculated by dividing tag counts for each gene with the total number of tags generated for each library and are presented per one million transcripts. PM and 1MM stand for perfect match and 1 miss match, respectively. (**A**) Mapping of total tags. (**B**) Mapping of distinct tags. Click here for file

Additional file 2: S2 Summary of tag mapping in DGE analysis for seven libraries. Click here for file

Additional file 4: S4 List of all genes identified by DGE. The first column represents names of the identified genes. Br_sense_raw and Br_antisense_raw mean the number of tags mapped to sense and antisense genes identified in DGE library of B. rapa. Br_sense_norm and Br_antisense_norm mean total times of detected tags per million in DGE library of *B. rapa*. GO Component, GO Function and GO Process mean the three main categories (cellular component, molecular function and biological process) in the GO classification, respectively. Click here for file

Additional file 5: S5 Sequencing saturation analysis of the seven libraries of *B. rapa* (Br), *B. oleracea* (Bo), *B. napus*-F_1_ (Bn-F_1_), *B. napus*-F_2_ (Bn-F_2_), *B. napus*-F_3_ (Bn-F_3_), *B. napus*-F_4_ (Bn-F_4_) and natural *B. napus* (Bn-N). The number of detected genes was enhanced as the sequencing amount (total tag number) increased. Click here for file

Additional file 6: S6 Distribution of ratio of distinct tag copy number in each pair of the libraries. ‘A’ was the control and ‘B’ was experimental group in ‘A vs. B’. Click here for file

Additional file 7: S7 List of differentially expressed genes in the early generations of resynthesized *B. napus*. DEGs between pairs of libraries were shown (Br vs. Bn-F_1_, Bo vs. Bn-F_1_, Bn-F_1_ vs. Bn-F_2_, Bn-F_1_ vs. Bn-F_3_, Bn-F_1_ vs. Bn-F_4_, Bn-N vs. Bn-F_1_. ‘A’ was the control and ‘B’ was experimental group in ‘A vs. B’). TPM: transcript copies per million tags. Raw intensity: the total number of tags sequenced for each gene. FDR: false discovery rate. We used FDR < 0.001 and the absolute value of log2Ratio ≤1 as the threshold to judge the significance of gene expression difference. In order to calculate the log2Ratio and FDR, we used TPM value of 0.001 instead of 0 for genes that do not express in one sample. Click here for file

Additional file 8: S8 The top 20 most up-regulated and down-regulated genes between samples (Br vs. Bn-F_1_, Bo vs. Bn-F_1_, Bn-F_1_ vs. Bn-F_2_, Bn-F_1_ vs. Bn-F_3_, Bn-F_1_ vs. Bn-F_4_, Bn-N vs. Bn-F_1_. ‘A’ was the control and ‘B’ was experimental group in ‘A vs. B’). TPM: transcript copies per million tags. This table does not include genes that only expressed in one sample. Click here for file

Additional file 9: S9 Histogram presentation of gene ontology classification of *B. rapa* (Br), *B. oleracea* (Bo), *B. napus*-F_1_ (Bn-F_1_), *B. napus*-F_2_ (Bn-F_2_), *B. napus*-F_3_ (Bn-F_3_), *B. napus*-F_4_ (Bn-F_4_) and natural *B. napus* (Bn-N). Click here for file

Additional file 10: S10 List of pathway enrichment analysis. Pathways with Q value ≤ 0.05 are significantly enriched in DEGs, see red-border region (‘A’ was the control and ‘B’ was experimental group in ‘A vs. B’). Click here for file

Additional file 11: S11 Both union and intersection DEGs used for HCE clustering analysis. Click here for file
