# Supplementary figures and images for: Use of digital gene expression to discriminate gene expression differences in early generations of resynthesized Brassica napus and its diploid progenitors
Source: BMC Genomics. 2013 Feb 1;14:72. doi: 10.1186/1471-2164-14-72 (PMC3608150; doi:10.1186/1471-2164-14-72)

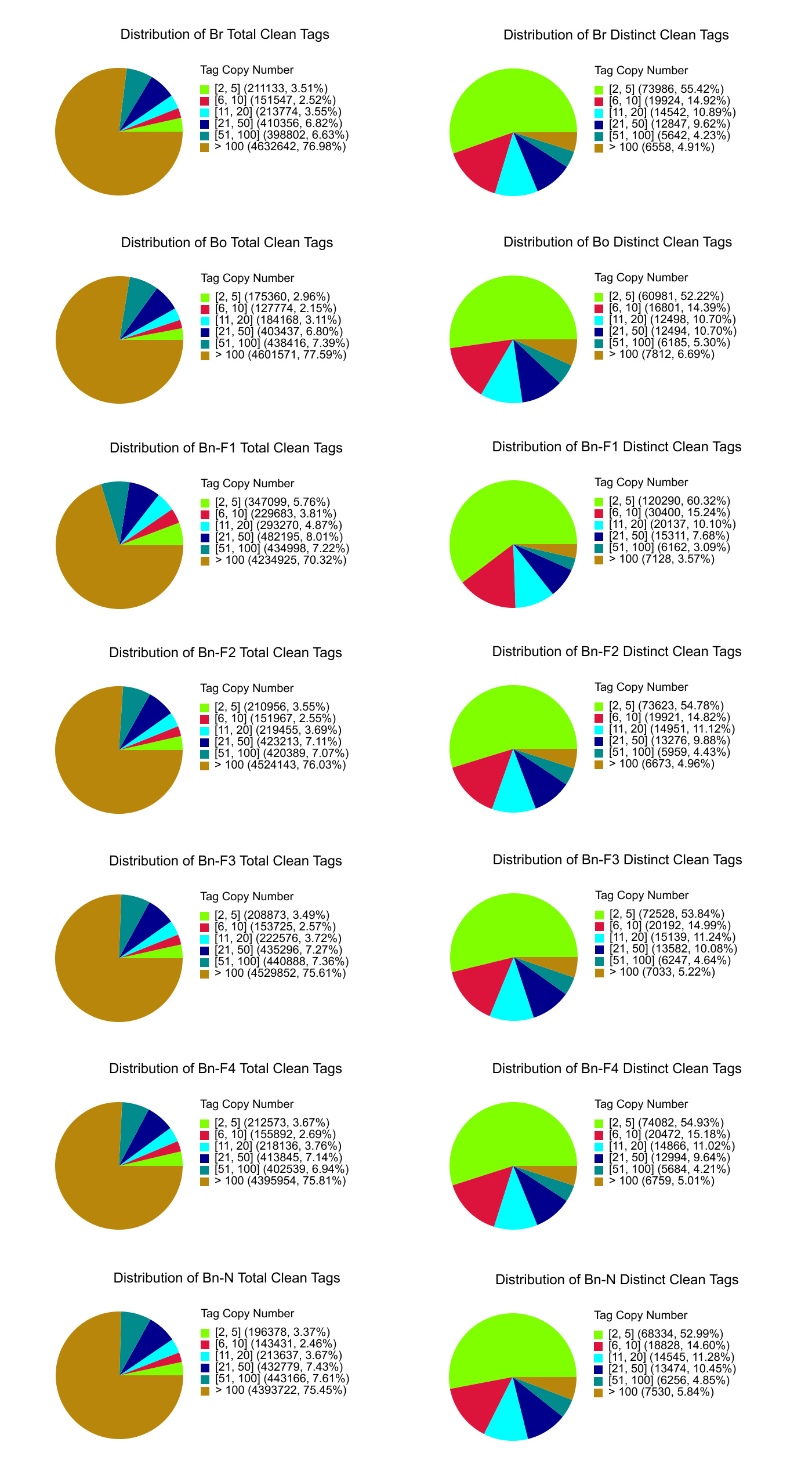

Supplement: Additional file 1: S1 — Distribution of total clean tags and distinct clean tags over different tag abundance categories in each DGE library. (A) Distribution of total tags. Numbers in the brackets of indicate the range of copy numbers for a specific category of tags. For example, [2,5] means all the tags in this category has 2 to 5 copies. Numbers in the parentheses show the total tag copy number for all the tags in that category. (B) Distribution of distinct tags. Numbers in the square brackets indicate the range of copy numbers for a specific category of tags. Numbers in the parentheses show the total types of tags in that category. [file 1471-2164-14-72-S1.jpeg]

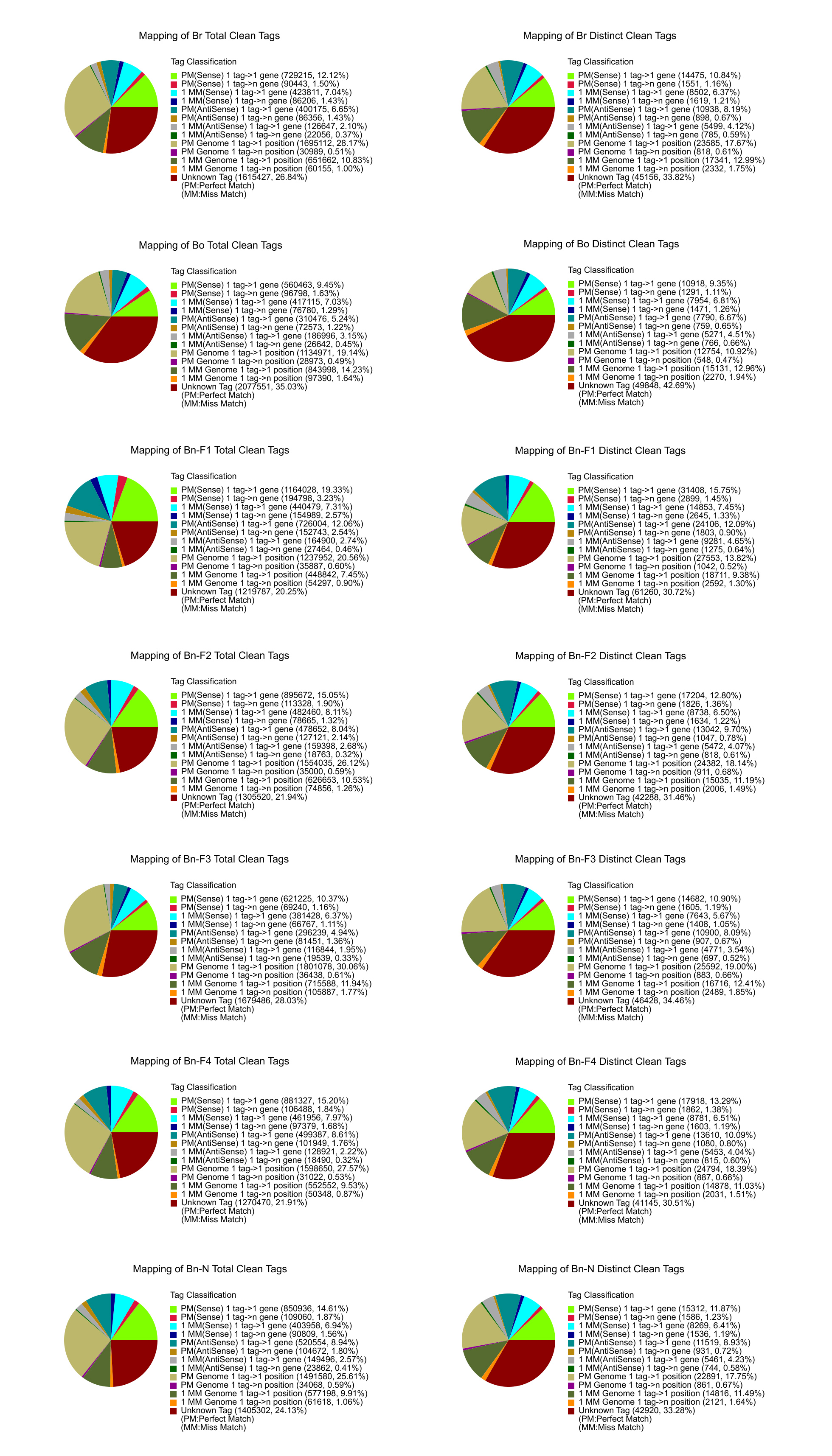

Supplement: Additional file 3: S3 — Mapping results of total tags and distinct tags of species in seven libraries. Normalized tag copy number was calculated by dividing tag counts for each gene with the total number of tags generated for each library and are presented per one million transcripts. PM and 1MM stand for perfect match and 1 miss match, respectively. (A) Mapping of total tags. (B) Mapping of distinct tags. [file 1471-2164-14-72-S3.jpeg]

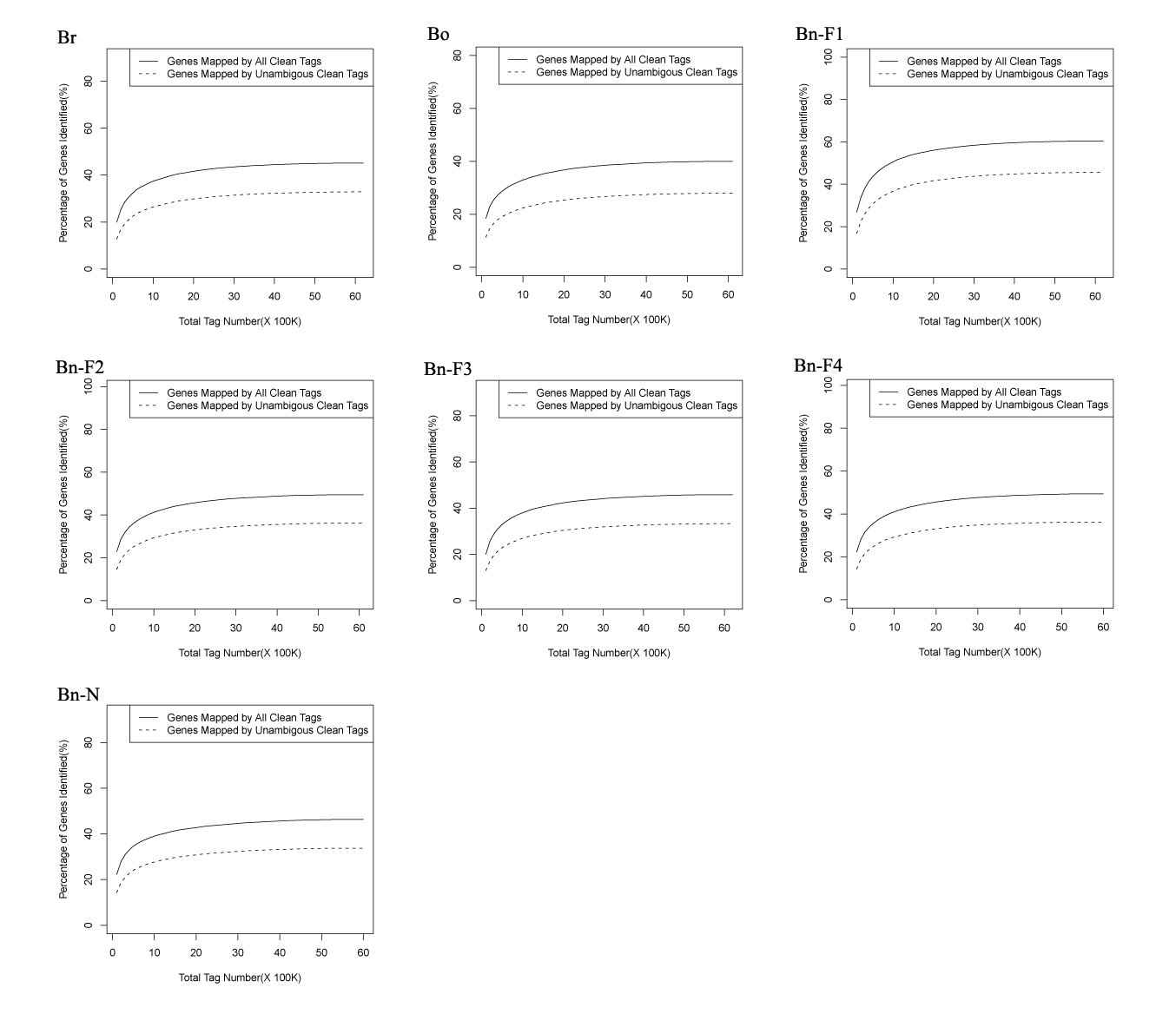

Supplement: Additional file 5: S5 — Sequencing saturation analysis of the seven libraries of B. rapa (Br), B. oleracea (Bo), B. napus-F1 (Bn-F1), B. napus-F2 (Bn-F2), B. napus-F3 (Bn-F3), B. napus-F4 (Bn-F4) and natural B. napus (Bn-N). The number of detected genes was enhanced as the sequencing amount (total tag number) increased. [file 1471-2164-14-72-S5.jpeg]

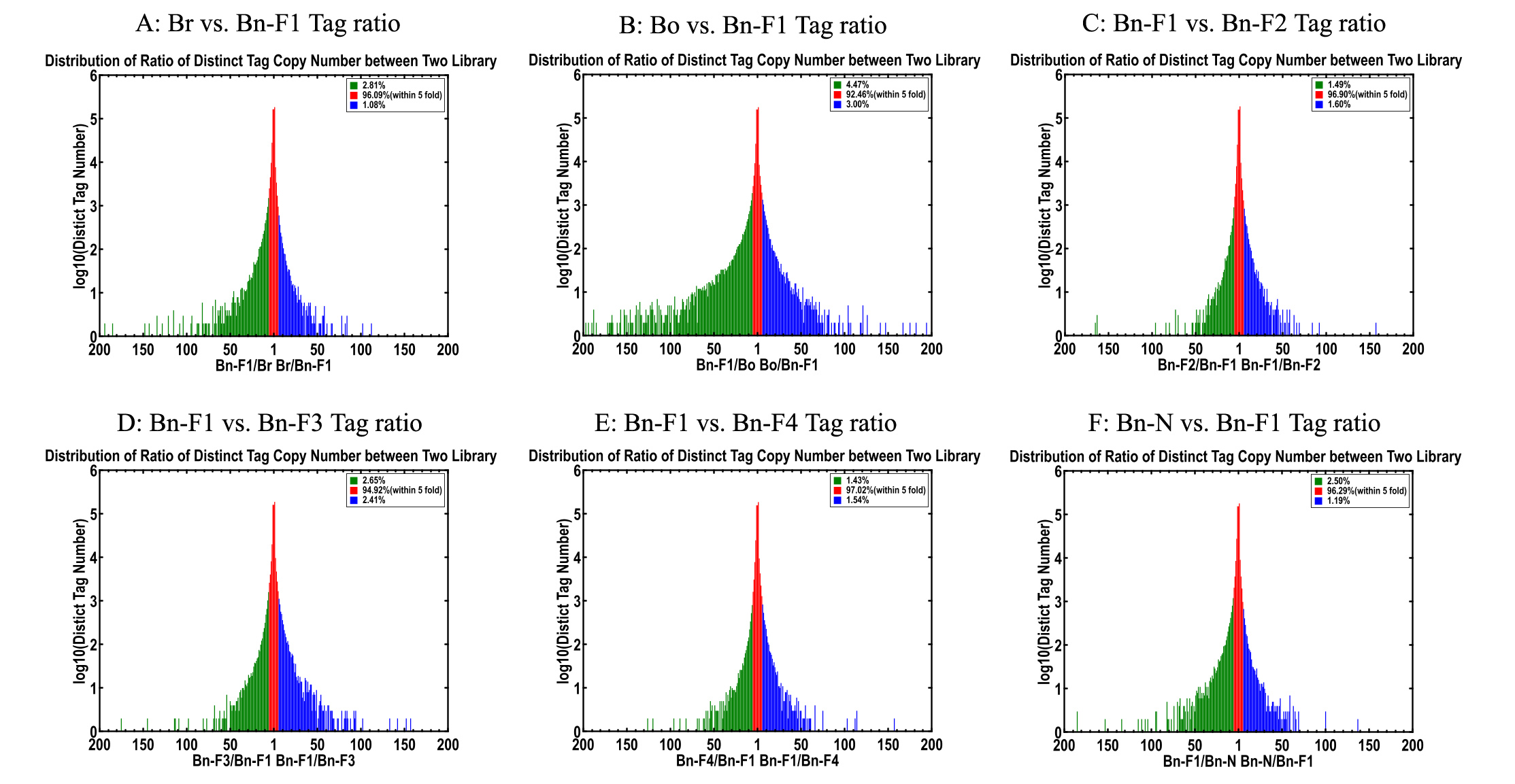

Supplement: Additional file 6: S6 — Distribution of ratio of distinct tag copy number in each pair of the libraries. ‘A’ was the control and ‘B’ was experimental group in ‘A vs. B’. [file 1471-2164-14-72-S6.jpeg]

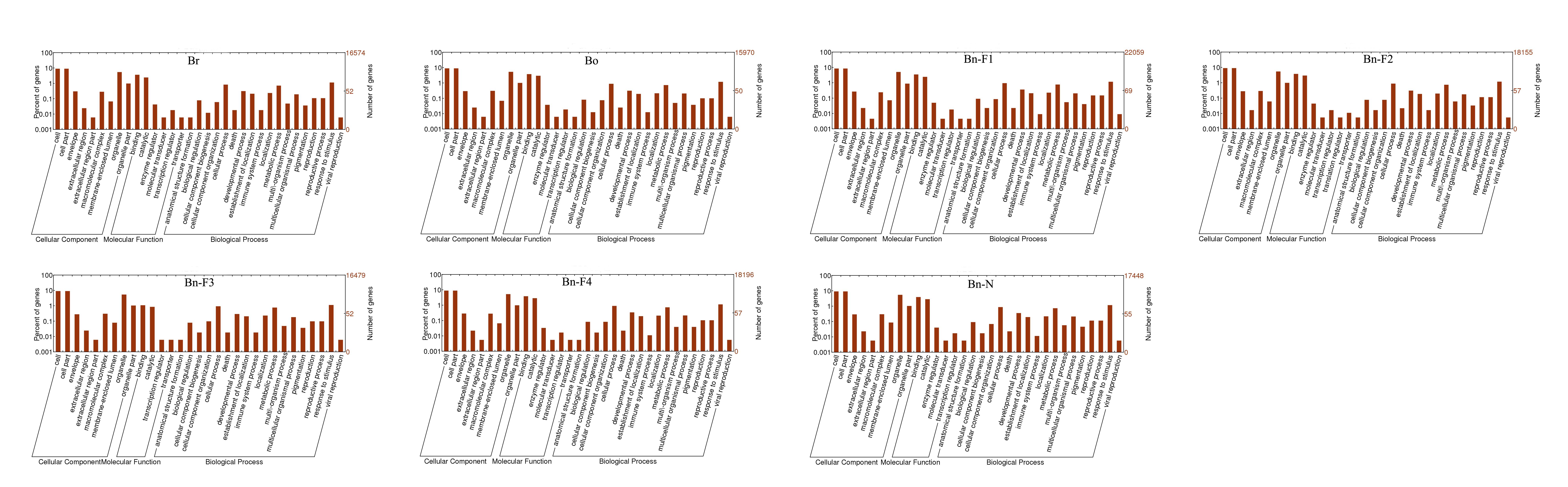

Supplement: Additional file 9: S9 — Histogram presentation of gene ontology classification of B. rapa (Br), B. oleracea (Bo), B. napus-F1 (Bn-F1), B. napus-F2 (Bn-F2), B. napus-F3 (Bn-F3), B. napus-F4 (Bn-F4) and natural B. napus (Bn-N). [file 1471-2164-14-72-S9.jpeg]
